# Cholera and Juggernaut

**Published:** 1858-10

**Authors:** 


Cholera and Juggernaut.

Dr. Nash, in a paper on Public Hygiene, published in the *Indian Annals of Medical Science* for 1858, adds a letter from the native medical subordinate of the Madras medical establishment, Mr. W. Vencataswamy Naidoo, on the causes which give rise to cholera amongst the pilgrims at Juggernauth. Having given an account of the sort and quality of the food prepared in the temple, and sold at enormous prices to the deluded pilgrims, the native writer, whose style we retain, relates thus:—

"People undergoing every fatigue and toil of long journeys, with privation of food and rest, arrive at their tabernacle half-starved or much exhausted, and have a small enclosed room pointed out by the pundahs for the accommodation of twenty or thirty persons; as soon as they have their baggage secured, they repair to the temple, washing themselves in different stinking ponds, and wearing wet or silk clothes (as an undefiled dress), visit the image, eat the various sorts of food with eagerness, taking no notice whatever of its condition, taste, or quality, under a well impressed idea that such observation would be an act of blasphemy, drink a jumboo-full of very sour rancid tyre, and feel themselves refreshed and very much satisfied for the first day, but from the second or third day the causes of diarrhœa, or cholera, well known to the medical world, viz., sudden transition from heat to cold, aliment of indigestible character, acrid food or acid drinks, oily and putrid substances, and want of free ventilation and drainage, soon operate upon the supposed repenters of sin.

"The streets and houses are impregnated with noxious exhalations emanated from the decomposition of the excrementitious and urinous deposits with which the streets, valleys, fields and plains are filled during the assemblage of people in great numbers, as well as from the dead bodies thrown out in the fields and in the towns.

"Cholera having thus originated, great alarm and despair are produced among the pilgrims, and the fright and despondency on one hand, and their longings for home, relatives or friends on the other, act conjointly as depressing agents, rendering their system more favourable to the action of the causes of cholera. The ravages made by this disease annually are very lamentable; and most pitiable is it to observe the annual scene at Juggernauth resulting from deaths in vast numbers, making children motherless or fatherless, or altogether orphans, women as widows, men as widowers, and afflicting others with the loss of brothers, sisters, etc. The loss of property is also effected, and many reach their native towns as beggars, being destitute of food, raiment, friends, relatives, money, or property. However much the deaths among the pilgrims may be ascribed to their moral depravity, still the deprivation of the sanitary reform so urgently needed at Juggernauth is a thing most deplorable."

